# Improved Method of Local Analgesia

**Published:** 1903-11

**Authors:** 


### ABSTRACTS.

---

### Improved Method of Local Analgesia.

ARTHUR E. J. BARKER, F. R. C. S., Eng. (*Lancet*, July 25, 1903), says he has employed local analgesia for surgical operations of considerable magnitude during the past five or six years, and has little doubt of its future when the principles which underlie it are well understood. The dangers associated with the injection of cocain appear to make it unsuitable for local analgesia in the larger and longer surgical operations. Prof. Barker has abandoned it in favor of beta-eucain, which is less dangerous and gives the most satisfactory results.

The great difficulty with local analgesia in extensive operations has been the inability to render the whole field analgesic, though up to three grains of beta-eucain (100 ccm. of a 2-1000 solution) may be injected, as ordinarily the effects of the injections pass off in about twenty minutes. Having recognised this and other drawbacks, he was very much interested in Dr. Braun's observations on a method of overcoming them by the combined use of beta-eucain with adrenalin. He has since used this method with great satisfaction. It is based on an old experience that anything which retards or diminishes the circulation of the blood in a part infiltrated with an analgesic, enhances the potency of the latter.

Prof. Barker prepares his analgesic solution as follows: 3 grains of beta-eucain and 12 grains of pure sodium chloride are added to 3½ fl. ozs. of boiling distilled water, and 16 drops of a 1-1000 solution of adrenalin chloride are poured into the fluid after it has cooled. This makes a normal saline solution containing 2-1000 eucain and 1-100,000 adrenalin chloride. The whole quantity could, within the limits of safety, be injected into a patient at one sitting; but Barker has found that an extensive operation can be done with about one-half of it.

The author then describes the radical cure of an inguinal hernia. Due regard must be taken of the position and course of the nerves supplying the structures to be dealt with, and in injecting around or into the nerves the aim should be to make each area of diffusion overlap the preceding one. Of course what is possible in the groin, which is proverbially sensitive, ought to be equally so in other parts.

Barker never observed secondary hemorrhage following the use of the method. He used it in 34 cases, which included 10 operations for radical cure of hernia, 5 cases of strangulated hernia, 2 castrations for tuberculous testis, 5 cases of varicose veins, 6 cases of psoas abscess, 1 case of loose body in the knee, 1 case of tumor of the neck (actinomycosis), 1 colotomy, 1 of Thiersch skin grafting, and 2 cystic adenoma of thyroid. The analgesia was always satisfactory and far exceeded in completeness and comfort all other procedures. Care must be taken to see that the adrenalin solution has not become inert by exposure to air or contamination with an alkali.

